# Demographics and Clinical Profiles of Patients Visiting a Free Clinic in Miami, Florida

**DOI:** 10.3389/fpubh.2019.00212

**Published:** 2019-08-02

**Authors:** Michael Zhang, Alejandro Garcia, Gisela Bretones

**Affiliations:** ^1^Miami Rescue Mission Clinic, Miami, FL, United States; ^2^School of Health Sciences, Miami Dade College, Miami, FL, United States

**Keywords:** public health, epidemiology, health policy, vulnerable populations, healthcare inequalities

## Abstract

**Background:** Although the ranks of the uninsured in the United States have decreased in recent years, some states still lack Medicaid expansion programs, leaving many Americans, especially the indigent and homeless, without adequate healthcare coverage. Free-for-care clinics are oftentimes the last safety net for these vulnerable populations. Because these clinics have limited funding, a thorough understanding of the patients they serve is necessary to effectively direct their resources. The objective of the present study is to investigate the characteristics and clinical profiles of patients utilizing a free clinic in Miami, Florida.

**Methods:** Aggregate EMR data reflecting consecutive adult patient visits to the Miami Rescue Mission Clinic in Miami, Florida between January 1st, 2018 to March 15th, 2019 (*n* = 846) were reviewed for sociodemographic characteristics and chronic disease prevalence. Prevalence rates were compared by sex and to county estimates from the Florida Behavioral Risk Factor Surveillance System.

**Results:** The most common conditions were mental health (19.3%), circulatory system (14.7%), and musculoskeletal system disorders (13.9%). Males had a greater prevalence of depression (difference = 6.6%; 95% CI [1.5 to 10.7%]; χ^2^ = 6.2; *p* = 0.013) and overall mental illness (22.0 vs. 10.4%, difference = 11.6%; 95% CI [5.7 to 16.4%]; χ^2^ = 13.2; *p* = 0.0003) compared to females, and male sex was identified as an independent risk factor for mental illness on multivariate logistic regression analysis (OR = 2.8; 95% CI [1.7 to 4.7]; *p* < 0.001). There was also a higher prevalence of depression (difference = 6.41%; 95% CI [2.1 to 10.2%]; χ^2^ = 8.0; *p* = 0.0047) and HIV (difference = 1.4%; 95% CI [0.3 to 3.0%]; χ^2^ = 7.3; *p* = 0.007) in male patients compared to county estimates. Rates of hypertension, diabetes, elevated cholesterol, asthma, and COPD were lower in the clinic population compared to the surrounding county.

**Conclusion:** There is an acute need for mental health services in this population. The lowered prevalence of other chronic conditions is due to underdiagnosis and loss to follow-up. Such analyses are important in guiding policy decisions for meeting the health needs of vulnerable, at risk populations.

## Introduction

The lack of health insurance is associated with numerous adverse clinical outcomes (1). Long recognized as a significant public health problem which affected a peak of 49.9 million Americans in 2010 (2), uninsurance has been linked with decreased health status (3), treatment delays (4), lower quality of care (5), underuse of preventive services (6), and increased overall mortality (7). Because of these consequences, there has been an increasing push for the adoption of universal health coverage (UHC) by a number of agencies, including the World Health Organization (WHO) (8) and the United Nations General Assembly (9). Nevertheless, the United States remains almost alone amongst developed nations in lacking a system of UHC.

To ameliorate some of these concerns, the Patient Protection and Affordable Care Act (ACA) was enacted in 2010 with the aim of improving healthcare access amongst previously uninsured Americans. By expanding access to Medicaid coverage and subsidizing private insurance premiums to adults with incomes below 138 and 400% of the federal poverty level (FPL), respectively (10), the ACA has reduced the ranks of the uninsured by some 43% five years after its implementation (11). However, these effects have not been uniform. Medicaid coverage expansion is an optional choice left to individual states (12), of which 14 have yet to adopt as of 2019. Although every state saw a reduction in uninsured rates, the greatest gains were seen in states which have adopted Medicaid expansion (13), and these differences in coverage rates have already translated into measurable differences in clinical outcomes (14).

Access to healthcare for low income patients in non-expansion states is limited. Although children, pregnant women, and parents may be eligible for Medicaid with FPL's varying from 18% to over 250% (15), other adults not in these specialized populations are not covered. This leaves out five million adults who would otherwise be eligible had their respective states participated in expanded Medicaid (16). One viable option for these patients is the utilization of free-for-care medical clinics (17). According to the American Medical Association (AMA), free clinics “provide services to low-income adults who are under- or uninsured (not covered by Medicare, Medicaid, or other government program) and are residents of the county in which the clinic is located” (18). Oftentimes supported through donations and staffed by volunteer providers, some 1,000 free clinics across the United States provide “a range of medical, dental or mental health services or medications directly to [the] economically disadvantaged” (19–21). Absent of other healthcare choices, these clinics play a crucial role as a last safety net for vulnerable populations (22–24). Because of the limited resources of these clinics, careful attention must be directed to the specific needs of their patients as well as the community in which they reside. Not only are there differences in the sociodemographic characteristics, environment, risk factors, health behaviors, and predictors of health between insured and uninsured adults (3), the uninsured themselves are not a homogenous group (25, 26). Therefore, it is crucial for free clinics to be familiar with and attentive to the characteristics of their particular clientele.

The purpose of the present study is to gain an understanding of the uninsured and indigent population of Miami-Dade County, Florida utilizing the services of a free clinic. Florida is one state without expanded Medicaid, leaving almost 900,000 residents without coverage (16). The clinic that is the focus of our study is the Miami Rescue Mission Clinic (MRMC) (27). First opened in 2009, the non-profit MRMC operates four separate facilities, with a main clinic in Miami-Dade and satellite clinics in both Miami and the surrounding county of Broward, all caring for indigent, homeless, and underserved members of the community. Offering primary care to adults and children, including patient visits, laboratory testing, diagnostic services, and medications, the MRMC also has partnerships with local hospitals for free specialty referrals. Supported through donations from individuals, local businesses, and organizations, the clinic's volunteer staff provided $1,935,243 in clinical services across 11,036 patient visits in 2017 alone. Much of this was directed to the local homeless community of Miami Dade. Indeed, the MRMC operates as part of the wider Miami Rescue Mission, an adjoining complex which provides a comprehensive residential program to the area's indigent population through food, shelter, education, and counseling, in addition to medical services. The present study investigates the demographics and health status of these patients, with particular attention to chronic diseases and mental illness, and with comparisons to estimates from the surrounding county.

## Materials and Methods

This study is a descriptive, retrospective review of aggregate electronic medical record (EMR) data reflecting consecutive adult patient visits for any reason from January 1st, 2018 to March 15th, 2019 to all branches of the Miami Rescue Mission Clinic. This study interval was selected because it denotes the time after which the current EMR system was fully implemented and its use mandated by the MRMC. Data were eligible for review if they contained at least one code from the Tenth Revision of the International Statistical Classification of Diseases and Related Health Problems (ICD10), and the patient was at least 18 years of age during the study period. All patient information was previously collected and diagnoses rendered by volunteer health care providers prior to study commencement. All data were retrieved from the EMR only on aggregate, i.e., the number of patients with such and such qualifier, rather than reviewing individual charts. We noted routine preexisting demographic data such as sex, race, ethnicity, and age distribution. Diagnosis codes were recorded and tabulated according to organ system and their prevalence calculated and sorted by sex. The prevalence of several specific conditions commonly encountered at MRMC, particularly chronic diseases and mental health, were further noted and compared with Miami-Dade County data from state and national databases. In particular, we used 2016 data from the Florida Behavioral Risk Factor Surveillance System (BRFSS), an ongoing and annual telephone-based survey under the direction of the Centers for Disease Control and Prevention (CDC) and administered by individual states. Most recently conducted in 2016, the Florida BRFSS “is the only source of state-specific, population-based estimates of the prevalence of various health conditions and related risk behaviors among Florida residents aged 18 and older” (28). Conducted in all 67 Florida counties, the survey contains questions on demographics, health status with respect to several chronic conditions, health related behaviors, and health related prevention. Miami Dade county data on HIV prevalence in the population over the age of 13 years were taken from the CDC's National Center for HIV/AIDS, Viral Hepatitis, STD, and TB Prevention (NCHHSTP) 2016 database (29). Statistical comparisons were performed using chi squared testing for proportions with one degree of freedom (df) with statistical significance at α = 0.05. Multivariable logistic regression analysis was used to test for independent demographic predictors of mental illness using categorical variables of sex, age group, race, and ethnicity, and data are reported as unstandardized regression coefficients (B) with standard errors (SE) and odds ratios (OR) with confidence intervals (CI). Statistical testing was conducted using IBM SPSS Statistics Version 24 for Windows (SPSS Inc., Chicago, Illinois) and JASP version 0.9.1.0, an open source software package (https://jasp-stats.org/). Ethical approval was not required for this study as it was a retrospective, non-interventional, non-observational review using existing data which were retrieved on aggregate and did not involve disclosure of protected health information (PHI). Individual patient charts were not accessed, and information pertaining to individual patients cannot be identified.

## Results

### Patient Demographics

A total of 846 patients met the inclusion criteria. These patients' demographics are summarized in (Table 1). A large majority (76%) of patients were male, reflecting the responsibility of MRMC as the entry point as well as the primary clinic for the adjoining Miami Rescue Mission's Center for Men, a “comprehensive, year-long residential regeneration Program for men who suffer from various life-controlling problems such as homelessness, chemical abuse or addiction, medical and mental issues, illiteracy, and lack of adequate job skills” (30). Of patients with known race, the majority were identified as Black or African-American (54.7%) or White (40.6%). Twelve percent of patients affirmatively declared their ethnicity as Hispanic, although 75.3% did not specifically indicate Hispanic status. Most patients were between 40–64 years of age, with a small minority of those aged 65 and over (7.7%).

**Table 1:** Patient demographics of our clinic population.

**Patient Demographics (*n* = 846)**	
**Sex**	
Male	645 (76.2%)
Female	201 (23.8%)
**Age (years)**	
18–39	36.3%
40–64	55.9%
65 and over	7.80%
**Race**	
American Indian	<1.00%
Asian	<1.00%
Black	54.7%
Hawaiian	<1.00%
White	40.6%
Declined	3.47%
**Ethnicity**	
Hispanic	12.0%
Not Hispanic	11.7%
Unknown	75.3%
Declined	<1.00%

### Prevalence of Medical Conditions by ICD10 Code

We examined the patients' diagnoses and classified them into systems by ICD10 code (Table 2). Overall, the most frequent conditions coded were mental and behavioral disorders (19.3% of all patients), diseases of the circulatory system (14.7%), and diseases of the musculoskeletal system and connective tissue (13.9%). Hypertension alone accounted for the overwhelming majority of circulatory system disorders (92%). Renal disorders and malignancy had a prevalence of <1%. When examined by sex, males had a greater prevalence of mental illness (difference = 11.6%; 95% CI [5.7 to 16.4%]; χ^2^ = 13.2; df = 1; *p* = 0.0003), nervous system disorders (difference = 4.8%; 95% CI [1.1 to 7.5%]; χ^2^ = 6.1; df = 1; *p* = 0.013), and dental disorders (difference = 3.1%; 95% CI [0.5 to 4.8%]; χ^2^ = 5.2; df = 1; *p* = 0.02), while females had a greater prevalence of genitourinary diagnoses, primarily urinary tract infections (difference = 3.9%; 95% CI [0.3 to 8.8%]; χ^2^ = 4.7; df = 1; *p* = 0.03).

**Table 2:** Prevalence of medical conditions by organ system, classified by ICD10 code.

**System**	**All patients *n* (%)**	**Males *n* (%)**	**Females *n* (%)**	**Sex difference *n* (%)**	***P*-value**
Circulatory	124 (14.7)	98 (15.2)	26 (12.9)		
Respiratory (Acute)	52 (6.15)	41 (6.36)	11 (5.47)		
Respiratory (Chronic)	38 (4.49)	33 (5.12)	5 (2.49)		
Gastrointestinal	61 (7.21)	51 (7.91)	10 (4.98)		
Genitourinary	46 (5.44)	29 (4.50)	17 (8.46)	3.96	0.0307
Endocrine	53 (6.26)	40 (6.20)	13 (6.47)		
Musculoskeletal	118 (13.9)	97 (15.0)	21 (10.4)		
Nervous	52 (6.15)	47 (7.29)	5 (2.49)	4.80	0.0134
Renal	1 (0.12)	0 (0.00)	1 (0.50)		
Eye	30 (3.54)	23 (3.57)	7 (3.48)		
Skin	57 (6.74)	47 (7.29)	10 (4.98)		
Teeth	24 (2.84)	23 (3.57)	1 (0.50)	3.07	0.0222
Malignancy	2 (0.24)	2 (0.31)	0 (0.00)		
Mental health	163 (19.3)	142 (22.0)	21 (10.4)	11.6	0.0003

### Prevalence of Mental and Behavioral Disorders

Because of the high prevalence of mental illness in our sample, we examined in greater detail the prevalence of individual disorders (Table 3). Overall, 19.3% of clinic patients were diagnosed with at least one mental health disorder. The most common condition diagnosed was any depressive disorder (12.5%) followed closely by any anxiety (11.8%). Bipolar disorder, schizophrenia, and other conditions, including substance abuse, post-traumatic stress disorder (PTSD), schizoaffective disorder, and attention deficit hyperactivity disorder (ADHD) each had a prevalence of less than 4%. When examined by sex, males had a greater prevalence of depression (difference = 6.6%; 95% CI [1.5 to 10.7%]; χ^2^ = 6.2; df = 1; *p* = 0.013), with no sex differences between the other conditions.

**Table 3:** Prevalence of psychiatric disorders by sex.

**Condition**	**All patients**	**Male *n* (%)**	**Female *n* (%)**	***P*-value**
Depression	106 (12.5)	91 (14.1)	15 (7.46)	0.013
Anxiety	100 (11.8)	84 (13.0)	16 (7.96)	
Bipolar disorder	30 (3.55)	26 (4.04)	4 (1.99)	
Schizophrenia	21 (2.48)	17 (2.64)	4 (1.99)	
Other	32 (3.78)	29 (4.50)	3 (1.49)	

*Other conditions include substance abuse, PTSD, schizoaffective disorder, and ADHD*.

### Independent Risk Factors of Mental and Behavioral Disorders

Multivariate binary logistic regression was performed to further explore the contribution of sex, age, race, and Hispanic ethnicity to mental illness in our clinic patients (Table 4). This analysis showed that only male sex was independently associated with an increased prevalence of any mental or behavioral disorder (OR = 2.8; 95% CI [1.7 to 4.7]; *p* < 0.001). The risks associated with Black race (OR = 1.4; 95% CI [0.9 to 2.1]; *p* = 0.07) and Hispanic ethnicity (OR = 1.6; 95% CI [0.9 to 2.8]; *p* = 0.08) did not quite reach statistical significance.

**Table 4:** Multivariate binary logistic regression analysis of demographic contributors to mental illness.

**Variable**	**B**	**SE**	**Wald**	**OR**	**95% CI**	***P-*value**
Sex (male vs. female)	1.04	0.26	15.7	2.82	1.69-4.71	<0.001
Age (years)						
18–39			1.63	Reference		0.44
40–64	−0.16	0.19	0.69	0.85	0.58–1.24	0.41
65 and over	0.25	0.38	0.42	1.28	0.61–2.70	0.52
Race (White vs. Black)	−0.37	0.19	3.28	0.71	0.49–1.03	0.07
Ethnicity (Hispanic vs. non-Hispanic)	0.48	0.28	3.00	1.64	0.94–2.77	0.08

### Selected Common Conditions With Comparisons to County Data

Finally, we analyzed the most common individual conditions diagnosed in clinic patients against the population of the surrounding county of Miami-Dade, which has a population of over 2.7 million in 2019 (Table 5). Compared to county estimates from the Florida BRFSS, our clinic sample had a lower prevalence of several chronic diseases (asthma, elevated cholesterol, chronic obstructive pulmonary disease (COPD), diabetes, and hypertension) in both males and females. However, the male prevalence of depression amongst clinic patients was significantly higher compared to male county estimates (difference = 6.4%; 95% CI [2.1 to 10.2%]; χ^2^ = 8.0; df = 1; *p* = 0.0047). Furthermore, the prevalence of HIV amongst both males (difference = 1.4%; 95% CI [0.3 to 3.0%]; χ^2^ = 7.3; df = 1; *p* = 0.007) and patients overall (difference = 1.5%; 95% CI [0.6 to 2.8%]; χ^2^ = 16.6; df = 1; *p* < 0.0001) were higher in the clinic sample compared to county surveillance data from the NCHHSTP, although there was no difference in females (difference = 0.4%; 95% CI [−0.3 to 3.0%]; χ^2^ = 0.6; df = 1; *p* = 0.4). When compared directly by sex, the prevalences of these conditions were not distinct, except for depression as noted above.

**Table 5:** Selected common conditions with comparison to county data from national databases.

**Condition**	**Clinic prevalence, both sexes (county prevalence)**	**Clinic prevalence, males (county prevalence)**	**Clinic prevalence, females (county prevalence)**
Asthma	2.72 (10.7)^a^	2.95 (10.9)^a^	1.99 (10.5)^a^
Elevated cholesterol	2.84 (27.8)^a^	2.32 (25.2)^a^	4.48 (30.1)^a^
COPD	1.65 (4.7)^a^	2.02 (5.2)^a^	0.50 (4.3)^a^
Depression	12.5 (11.7)	14.1 (7.7)^b^	7.46 (25.7)^a^
Diabetes	4.85 (9.2)^a^	5.12 (9.8)^a^	3.98 (8.5)^a^
HIV	2.60 (1.1)^b^	3.10 (1.7)^b^	1.00 (0.6)
Hypertension	13.5 (32.7)^a^	14.0 (32.7)^a^	11.9 (32.7)^a^

a *Prevalence in clinic sample less than county prevalence*.

b *Prevalence in clinic sample greater than county prevalence*.

## Discussion

Multiple studies have previously investigated the demographics and clinical characteristics of patients utilizing local free clinics in other metropolitan settings (31–38). Common clinical findings across these studies include a higher prevalence of smoking (35, 36), obesity (31, 35, 36), hypertension, asthma, and diabetes (31) amongst this population compared to county or national estimates. Mental health disorders were also noted at increased rates in several studies (31, 34, 36). In our study of patients utilizing free medical services of our clinics in Miami-Dade and the adjacent county of Broward in Florida, we found that male patients had a higher prevalence of depression compared to both female patients and the general population of the surrounding county. Furthermore, our patients had a higher prevalence of HIV infection. The prevalence of other medical conditions, particularly hypertension, dyslipidemia, diabetes, asthma, and COPD, were lower than county estimates.

The lowered rates of these chronic conditions were almost certainly due to underdiagnosis. This is a common problem amongst the uninsured (7) and leads to delayed diagnosis (39) and increased disease severity upon eventual diagnosis (40). Of course, the development of a comprehensive clinical assessment requires compliance with consistent visits, follow ups, laboratory appointments, and referrals for specialty evaluations. Because of the inherently transient nature of the indigent and homeless population who utilize our clinic, many patients cannot adhere to these commitments and are simply lost to follow up. Indeed, the average number of visits per patient was only around 2.6, with a third of the patients in our sample seen only once and never again. All of these factors can lead to worse clinical outcomes (40).

It is noteworthy that the prevalence of mental illness was nevertheless elevated despite this overall underdiagnosis. This should draw attention to the acute need for mental health services for indigent populations, particularly since they are less likely to receive treatment (41). In our study population, we found that the most prevalent mental health disorders were depression and anxiety, with the former substantially exceeding county estimates in males (14.1 vs. 12.5%, *p* = 0.0047). Furthermore, while county level data were not available, our patients appeared to have higher rates of both bipolar disorder (3.5 vs. 2–3%) (42–44) and schizophrenia (2.5 vs. 0.4%) (45) compared to national estimates in the general population. These findings agree with previous studies that have indeed suggested that the overall rate of psychiatric disorders is elevated amongst both the uninsured (46) as well as the homeless (47, 48). Conversely, the mentally ill are also more likely to be uninsured (49, 50) and homeless (51). Furthermore, rates of individual disorders are higher in the latter population. However, one study attributes this largely to their increased prevalence of alcohol and drug abuse (48), in contrast to the predominance of depression and anxiety in our clinic patients. Nevertheless, it is overall apparent that more resources should be directed toward the provision of mental health services to this population.

The sex distribution of mental disorders in our sample is interesting and offers further insight into the crucial role of regular follow-up in the establishment of proper diagnoses. It has been reported that the prevalence of both chronic mental (26, 52) and physical (26) disorders are heighted amongst the female homeless compared to males. Even in Miami-Dade County, the lifetime prevalence of depression in women (25.7%) greatly exceeds that in men (7.7%). However, we found a significantly greater rate of mental illness in our male clinic population compared to females (22.0 vs. 10.4%, *p* = 0.0003). One possible reason for this skew is a difference in diagnostic opportunities for females. Although the average visit number for all patients in our sample was 2.6, there was a stark difference between the number of visits for men (mean 2.9, SD 2.1) and women (mean 1.6, SD 1.3) (*p* = 0.0002). As mentioned previously, one of the functions of our clinic is to provide initial and ongoing care to clients of the adjoining men's shelter at MRC. The residential programs therein provide not only food and shelter, but ongoing training and educational opportunities which promote reintegration into society, thus encouraging long term stay. This provides a degree of domestic stability that appears to translate into more regular clinic visits, with increased opportunities for diagnosis, treatment, and outside referral for specialized mental health care. While women are welcome at our clinic, they are generally from further away in the surrounding community, and this lack of proximity may act as a barrier to ongoing care. Therefore, it is not surprising that the prevalence of both mental and physical diagnoses is generally equal or lower amongst women in our clinic sample, as it is merely an expression of the general rule that reliable diagnosis depends on regular and consistent follow-up.

The high prevalence of HIV in this sample is unsurprising. The burden of HIV disproportionally affects indigent populations (53) and is associated with underdiagnosis (54), poor adherence to medications (55), incomplete viral suppression (56), higher rates of comorbidities (57), and early death (58). Furthermore, homelessness itself is associated with risky behaviors that increase the probability of disease transmission (59). Studies have suggested that housing intervention in this population may yield beneficial outcomes (60–63) and governmental efforts though the Housing Opportunities for Persons with AIDS (HOPWA) Program are aimed at meeting the housing needs of these patients. However, funding may be an issue, and Miami's HOPWA program is currently not accepting applications. The provision of community based housing (64) may offer additional options to the homeless population with HIV.

This study has several limitations. As mentioned previously, underdiagnosis due to inadequate follow up is a concern in this transient population. However, clinic characteristics may also play a role. Because the clinic is staffed by a number of volunteer providers, there may be variances in diagnosis and documentation due to individual provider preferences. For example, some providers may not explicitly enter ICD10 codes into the record system, preferring instead to simply type diagnoses in their notes. To avoid this from causing apparent underdiagnosis, patients without any ICD10 codes were entirely excluded from this study. This should not have caused the opposite problem of apparent overdiagnosis in the remaining patients, as healthy patients visits were generally associated with Z00^*^ codes. However, concerns may yet be raised about possible overdiagnosis of mental illness amongst this population. This is not an unrecognized problem in the homeless, and it has been suggested that this may be due to racial differences (65, 66). As noted before, we did not demonstrate race as an independent risk factor for mental illness, and our findings are consistent with other studies which demonstrate a disproportionate skew toward mental illness vs common medical conditions such as hypertension and diabetes in the homeless (67, 68). Further, the low prevalence of “other” mental health disorders, particularly substance abuse, is almost certainly an artifact. The rates of alcohol and drug dependence are known to be elevated in homeless populations (48), and their low apparent prevalence in our study is probably due to a combination of patient underreporting along with other patient and clinic characteristics as suggested above. Finally, discordances in methodology between clinical diagnosis and BRFSS inquiry may make prevalence comparisons difficult. For example, BRFSS questions usually take the form of “Have you ever been told by a doctor, nurse, or other health professional that you have such and such condition?” Of course, this is not the same as actually being diagnosed with the said condition in the clinical setting and is a limitation of the retrospective study design. The use of prospective surveys with similar questions to BRFSS questionnaires would be a more reliable method for direct prevalence comparisons and may also address apparent underdiagnosis.

## Conclusion

There is a significant population of vulnerable patients that remain without health insurance coverage in states without expanded Medicaid. Healthcare options for these patients are limited, and community based free-for-care clinics may serve as their last safety net. While this population is not homogenous, many are homeless, and their social characteristics are inextricably intertwined with their health characteristics. A thorough understanding of both is necessary for free clinics to provide an optimal level of care to these patients, and particular attention should be directed to the provision of mental health services.

## Data Availability

All datasets analyzed for this study are included in the manuscript.

## Author Contributions

MZ contributed to study conceptualization, design, data collection and interpretation, and manuscript writing. AG and GB contributed to design, data interpretation and manuscript editing. All authors reviewed and approved the final manuscript.

### Conflict of Interest Statement

The authors declare that the research was conducted in the absence of any commercial or financial relationships that could be construed as a potential conflict of interest.
